# Notes and Comments

**Published:** 1895-03

**Authors:** 


### Notes and Comments.[1]

True Culture.—It is not always the men or women who are acknowledged leaders in society, nor those who are masters of any special science, or can best master a college curriculum, who are the most cultured, but those who are masters of themselves, without bias or bigotry, ready to fulfil the duties of life with an earnest purpose to make the most of themselves and of the society of which they are a part.

True culture is the enlargement of our faculties, with an aim to make life fuller of all that is true, good, and useful.

---

The Application of Chloride of Ethyl.—A contributor to the *Lancet* calls attention to the fact that the vapor of ethyl chloride, when inhaled, is not free from injury. He cites a case where, after applying the liquid for a short time, "the patient stopped breathing, turned pale, slightly livid, looking very like a person under nitrous oxide gas." The writer then states that he will certainly not use it again where it is possible that the vapor can be inhaled.

We have had a large clinical experience with ethyl chloride, having employed it as a local anæsthetic since it was first placed upon the market, and while in a few cases, where the vapor was freely inhaled, symptoms of general anæsthesia were shown,—the effects of the chlorine,—nothing in the least alarming has been seen. We soon learned that by directing the patient to breathe entirely through the nose, and protecting this organ by bringing the edge of the napkin in front of it, this unpleasant feature was overcome. We feel no hesitancy in saying that for all minor surgical operations, especially such as the dentist is called upon to perform, chloride of ethyl is the safest and most efficient local anæsthetic extant.

Dr. Louis Jack, in writing us upon the subject, says, "I have found by experience that it is superior for producing refrigeration in extracting teeth and roots, and in implantation, to the usual modes of inducing local anæsthesia by ether or rhigolene."

---

[1] The assistant editor solicits contributions for this department,—new methods, new remedies and formulas, or any short practical note which may prove of value to the practitioner or student. Address 1718 Walnut Street, Philadelphia.

